# Hearing Norton Sound: community involvement in the design of a mixed methods community randomized trial in 15 Alaska Native communities

**DOI:** 10.1186/s40900-020-00235-0

**Published:** 2020-11-03

**Authors:** Samantha Kleindienst Robler, S. Meade Inglis, Joseph J. Gallo, Heather E. Parnell, Paul Ivanoff, Stephanie Ryan, Cole D. Jenson, Alexandra Ross, Alain Labrique, Nae-Yuh Wang, Susan D. Emmett

**Affiliations:** 1 Department of Audiology, Norton Sound Health Corporation, Nome, AK USA; 2 Center for Health Policy and Inequalities Research, Duke University, Durham, NC USA; 3 Duke Global Health Institute, Durham, NC USA; 4 Department of Mental Health, Johns Hopkins Bloomberg School of Public Health, Baltimore, MD USA; 5 Hearing Norton Sound, Unalakleet, AK USA; 6 Hearing Norton Sound, Anchorage, AK USA; 7 Department of Head and Neck Surgery and Communication Sciences, Duke University School of Medicine, Durham, NC USA; 8 Department of International Health, Johns Hopkins Bloomberg School of Public Health, Baltimore, MD USA; 9 Department of Medicine, Johns Hopkins University School of Medicine, Baltimore, MD USA; 10 Departments of Biostatistics and Epidemiology, Johns Hopkins Bloomberg School of Public Health, Baltimore, MD USA

**Keywords:** Indigenous circumpolar health, Stakeholder involvement, Community participation, Community engagement, Randomized controlled trial, Community-based research

## Abstract

**Plain English summary:**

Community involvement is important in good research practice. We led a community-based study to improve early detection and treatment of childhood hearing loss in rural Alaska. This study evaluated a cell phone-based hearing screening process and compared a new telemedicine specialty referral pathway to the standard primary care referral pathway. The study included community involvement, engagement, and participation from the very beginning to inform how to best design the trial. We obtained insight and feedback from community members through involvement of a core stakeholder team and through community engagement and participation in focus groups and community events. Feedback received through community involvement and participation influenced the design of the trial at key decision points. Community member guidance shaped the research question, the outcomes to be measured, and the procedures for completing the project, such as participant recruitment. This study offers an example of community involvement, engagement and participation that could be mirrored in future research to maintain the interests of participating communities.

**Abstract:**

**Background**

Effective systems for early identification and treatment of childhood hearing loss are essential in rural Alaska, where data indicate a high prevalence of childhood ear infections and hearing loss. However, loss to follow-up from school hearing screening programs is pervasive. The Hearing Norton Sound study was a mixed methods community randomized controlled trial that was developed to address this gap. The study engaged community members and participants in the design of the trial, including involvement of stakeholders as collaborators.

**Methods**

Community engagement and participation in research design occurred through focus groups and through the integration of stakeholders into the study team. Representation was cross-sectoral, involving individuals from multiple levels of the school and health system, as well as community members from each of the 15 communities. Feedback obtained between April 2017 and August 2017 informed the final design of the randomized trial, which began enrollment of children in October 2017 and concluded in March 2019.

**Results**

Stakeholder involvement and community participation shaped the design of specific trial elements (research question; comparators; outcomes and measures; telemedicine protocols; and recruitment and retention). Community involvement was strengthened by the use of multiple modalities of involvement and by the positionality of lead stakeholders on the study team.

**Conclusions**

This study highlights the effectiveness of multifaceted stakeholder involvement and participation in the design of health research conducted within Alaska Native communities. It offers an example of involvement and reporting that could be mirrored in future research in order to protect and further the interests of the participating community.

**Trial registration**

ClinicalTrials.gov, NCT03309553, First registered 10/9/2017

## Background

Childhood hearing loss can have profound, lifelong consequences on speech and language development, school achievement, future employment opportunities, and quality of life [1–6]. Some regions experience a disproportionate burden of childhood hearing loss. In rural Alaska, historical data indicate a high prevalence of ear infections and subsequent hearing loss [7–9]. Effective systems for early identification and treatment of childhood hearing loss are therefore essential in rural Alaska.

**Fig. 1:**
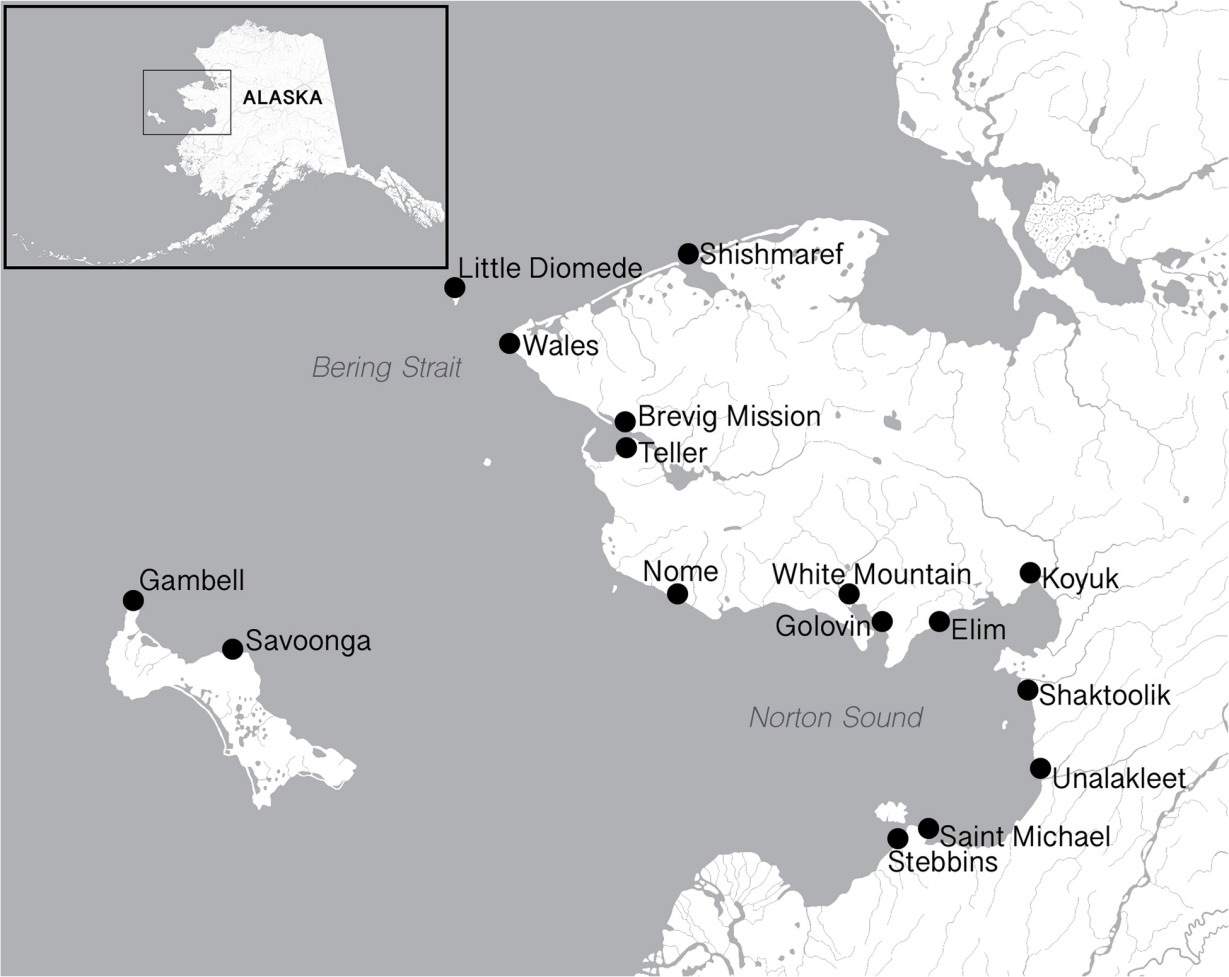
Map of communities in the Bering Strait region in Northwest Alaska

The Hearing Norton Sound study, a mixed methods community randomized trial, evaluated the effectiveness of a cell phone-based (mHealth) school hearing screening and telemedicine specialty referral pathway for improving timely identification and treatment of childhood hearing loss. Results from the trial are not yet available, but detailed protocols are published [10, 11]. The study included 15 rural Alaska Native communities across the Bering Strait region, which spans approximately 23,000 square miles in Northwest Alaska (see Fig. 1). The Central Yup’ik communities of Stebbins and St. Michael mark the southernmost points of the region. Three island communities mark the Western edge of the region: the St. Lawrence Yu’pik communities of Gambell and Savoonga, and the Iñupiaq community of Little Diomede. The Northern communities of Teller, Brevig Mission, Wales and Shishmaref line the Bering Strait coastline and are home to primarily Iñupiaq Eskimos. The communities of Unalakleet, Shaktoolik, Koyuk, Elim, Golovin and White Mountain surround the coastline of the Norton Sound and are home to Yup’ik, Iñupiaq and Athabascan peoples. Nome, the largest community in the region, is considered the regional hub.

Norton Sound Health Corporation (NSHC), a tribally-owned nonprofit health system, is based in Nome and serves the region’s 15 rural communities. These communities are accessible almost exclusively by plane or helicopter, making the delivery of specialty health care services complex. NSHC has adopted a robust model of telemedicine, in which trained community health aides/practitioners (CHA/Ps) provide frontline care in community clinics, using telemedicine capabilities to consult primary care physicians and specialists based in Nome and Anchorage for diagnosis and treatment plans. Formal training for CHA/Ps began in the 1960’s and has resulted in a large network of CHA/Ps working alongside licensed providers to offer increased access to care across rural Alaska [12, 13]. For ear and hearing-related cases, telemedicine has been regionally adopted as the standard of care, and validation studies have demonstrated that medical decision-making through telemedicine is equivalent to an in-person exam [14–16].

The Bering Strait School District (BSSD) serves the region’s 15 communities. BSSD facilitates early identification and treatment of childhood hearing loss by conducting annual school hearing screenings, which are mandated by the state of Alaska [17]. While not formally measured before the initiation of the Hearing Norton Sound study, a large percentage of children are typically referred for follow-up after school screening each year due to the high prevalence of ear infections and hearing loss in rural Alaska. However, there are significant challenges to providing the necessary follow-up care, and health care providers and school staff have anecdotally observed substantial loss to follow-up for referred children.

The Hearing Norton Sound study was developed in collaboration with stakeholders to address loss to follow-up from school hearing screening, with the goal of developing a system for timely identification and treatment of childhood hearing loss. By involving stakeholders as collaborators and community members through engagement and participation, the Hearing Norton Sound study prioritized the preferences of stakeholders in an effort to protect, preserve, and further their interests. Community activities in research can take on several forms that are distinct but complementary: *community involvement* as active partnership between community stakeholders and researchers in the research process; *community participation* as enrollment in study activities, such as focus groups; and *community engagement* as dissemination of information related to the study [18, 19]. Through each of these domains, the study team sought stakeholders’ perspectives on hearing loss and the approach of the research project. These processes are consistent with broad recommendations to maximize the use of qualitative methodologies within mixed methods trials [20].

In health research with Alaska Native communities, such collaborative and community-based processes are encouraged, if not required, as ‘best practices’ [21, 22]. However, few publications describe the processes of involvement within indigenous communities in the Circumpolar North [23–25]. This is consistent with a generalized paucity of reporting on stakeholder involvement in research [26]. The aim of this paper is to clearly describe how stakeholder involvement and participation influenced the design of this community randomized trial.

## Methods

### Overview

The Hearing Norton Sound study began with an exploratory sequential stage, followed by an explanatory sequential stage [11]. The processes for community involvement and participation described within this paper occurred during project planning stages and the exploratory sequential stage. The study was reviewed and approved by the Institutional Review Boards of Alaska Area, Norton Sound Health Corporation, and Duke University.

**Fig. 2:**
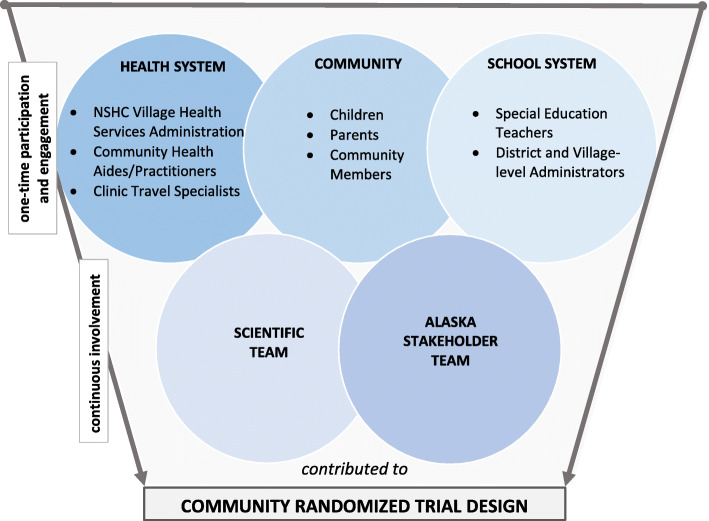
Through focus groups and other one-time mediums of participation (meetings, phone calls, emails), stakeholders in the communities, the health system, and the school system who were not formally a part of the study team contributed to the design of the community randomized trial. Lead stakeholders from each of these sectors were embedded within the Alaska-based stakeholder team to facilitate continuous involvement. For the full description of members of the Hearing Norton Sound scientific team and the Alaska-based stakeholder team, see Table 1

Involved individuals were members of the 15 communities and stakeholders within the healthcare and education sectors (Fig. 2). The intent of this cross-sectoral involvement and participation was to adopt an ecological approach which considered the systemic, social, and environmental factors relevant to the identification and treatment of childhood hearing loss [27, 28]. Formats for involvement, engagement, and participation were diverse. Community members and stakeholders from each sector were involved in one-time focus groups and community events, as well as on-going or continuous exchange through meetings, phone calls, video conferences, and emails. Invitations to participate in focus groups as well as community events for engagement were posted through flyers, radio, and social media announcements, in accordance with local recommendations. Lead stakeholders from each involved sector were identified through the local social network of the Lead Audiology Stakeholder. They had diverse experiences with the local school and health systems, and several had personal experiences with hearing loss and ear infections. These stakeholders became the Alaska stakeholder team and contributed valuable cultural insight as community members, as consumers of their own healthcare, and as parents. The Alaska stakeholder team worked in partnership with the scientific team to form the complete Hearing Norton Sound study team (Table 1).

**Table 1:** Overview of the multidisciplinary Hearing Norton Sound study team

Study Team			
Scientific Team		Alaska Stakeholder Team	
Position	Background	Position	Background and positionality
Principal Investigator	- Otolaryngologist - Practicing surgeon - Public health researcher with expertise in hearing loss disparities	Principal Investigator and Lead Audiology Stakeholder	- Director of Audiology at NSHC - Practicing audiologist - Clinical researcher - Lived in and served region for 9 years
Statistician	- Statistician with expertise in randomized trials	Lead Hospital Administration Stakeholder	- Vice President of Hospital Services for NSHC - Audiologist and former Director of Audiology at NSHC - Lived in and served region for 20 years
Mixed Methods Co-Investigator	- Physician - Public health researcher with expertise in mixed methods	Lead Parent Stakeholder	- Alaska Native, parent, patient, professional, resident of one of the 15 participating communities
mHealth Co-Investigator	- Public health researcher with expertise on technology integration in randomized trials	Communications Outreach Specialist	- Alaska Native, parent, patient, journalist, resident of one of the 15 participating communities
		Lead Patient Partner	- Alaska Native, parent, patient, originally from one of the 15 participating communities
		Lead Education Stakeholder	- Coordinator of Special Education for Bering Strait School District, provides oversight for Special Education teachers conducting school hearing screenings, - Longtime resident of one of the 15 participating communities
		Lead Surgeon Stakeholder	Otolaryngologist practicing within the state of Alaska, with expertise in telemedicine

*NSHC* Norton Sound Health Corporation

### Focus group participation and engagement

Eleven focus groups were hosted in a six-month period leading up to the randomized trial, with representation from each of the region’s 15 communities. Six of the eleven focus groups were considered community events and were open to all community members to facilitate engagement and obtain feedback from the community at large. The remaining five focus groups were stakeholder-specific and dedicated to the participation of teachers, parents, and community health aides. Two of these stakeholder-specific focus groups were hosted via video conference, which allowed for wider participation across the remote communities. Focus group size ranged from 11 to 29 participants, with a total of 116 consented participants across the 11 focus groups. Participant ages ranged from 15 to 87 years, and many participants described personal experiences with hearing loss and ear infections. Focus group discussions were based on a combination of information sharing and awareness, as well as a predetermined interview guide with open-ended questions. Written informed consent was obtained from all participants prior to participation. The focus groups were facilitated by at least two trained moderators: Lead Parent Stakeholder, Communications Outreach Specialist, Lead Audiology Stakeholder, and/or the Lead Hospital Administration Stakeholder. All moderators were either from the region or well known in the region, with at least one Alaska Native moderator at all events to facilitate trust and culturally relevant dialog. Focus groups were audio-recorded and ranged in duration from 60 to 120 min. A technological error caused the loss of one focus group audio recording. The facilitators noticed this immediately and took detailed notes on the discussion.

### Stakeholder team involvement

The Alaska stakeholder team was involved through phone calls, emails, video conferences, and meetings. These interactions allowed for continuous guidance and insight that supported the one-time feedback received from focus groups and community events (Fig. 2). The flexibility of these formats, including both the one-time focus groups and the ongoing involvement of the Alaska stakeholder team, enabled iterative feedback through the course of the project.

### Data analysis

All focus group audio recordings were transcribed verbatim, and summary notes were made. Identifying information was removed from transcripts and notes prior to analyses. All transcripts and notes were double-coded with QSR International NVivo® 11 by two independent coders. Originally developed for use in the grounded theory method of Glaser and Strauss [29], coders applied the constant comparative method by taking one theme and comparing it with others that may be similar or different [30]. Regular meetings were held with the scientific and Alaska stakeholder teams during analysis, and the Lead Parent Stakeholder, Lead Patient Partner, and the Communications Outreach Specialist were integrally involved in reviewing content and the themes derived from that content. Specifically, the Lead Parent Stakeholder, Lead Patient Partner, and the Communications Outreach Specialist facilitated interpretation of community event and focus group themes for translation into study design.

For the continuous and dynamic mediums of involvement, (e.g. meetings, phone calls, emails, video conferences), process notes were recorded. These notes, along with consultation with the Alaska stakeholder team, informed the processes for involvement presented here.

## Results

Descriptions of community participation, engagement, and involvement in each area of trial design for the Hearing Norton Sound community randomized trial can be found in Table 2. We illustrate the stakeholder groups involved with descriptions of involvement for each aspect of research design. A reporting checklist following the Guidance for Reporting Involvement of Patients and Public (GRIPP2) Long Form can be found in Additional file 1 [26].

**Table 2:** Descriptions of stakeholder engagement, participation, and involvement in each trial design area, Hearing Norton Sound community randomized trial (2017–2020)

Design Area	Groups Involved	Descriptions of Involvement
**Research question development**	-Lead Audiology Stakeholder -Lead Surgeon Stakeholder -Lead Hospital Administration Stakeholder -Lead Education Stakeholder -Communications Outreach Specialist -Lead Parent Stakeholder -Scientific Team	• Observed systemic weaknesses in the processes of identifying and treating children with hearing loss. • Agreed upon the legitimacy of researching an intervention. • Identified school hearing screenings and referrals as processes to measure.
**Comparators for screening processes**	-Lead Education Stakeholder -Teachers	• Requested that the selected intervention screening process prioritize affordability and ease of use.
**Comparators for referral processes**	-Scientific Team -Alaska Stakeholder Team	• Identified potential weaknesses of the standard primary care referral pathway, built intervention telemedicine specialty referral pathway.
**Unit of Randomization**	-Scientific Team	• Proposed randomization.
	-Lead Audiology Stakeholder -Lead Surgeon Stakeholder -Communications Outreach Specialist -Lead Parent Stakeholder -Lead Patient Partner	• Advocated for screening processes to not be randomized, but standardized for all participants. Advocated for referral pathways to be randomized at the community, not individual, level.
	-Communications Outreach Specialist -Lead Parent Stakeholder	• Guided scientific team through forming strata for referral pathway randomization based on geographic and sociocultural considerations across the 15 participating communities.
**Choice of Outcomes & Measures**	-Lead Hospital Administration Stakeholder -Lead Audiology Stakeholder -Lead Surgeon Stakeholder -Scientific Team	• Determined measurements for the primary outcome and some of secondary outcomes, including sensitivity and specificity of the school and mHealth screenings.
	-Lead Education Stakeholder	• Directed scientific team to use AIMSweb test scores, the standard for academic benchmark assessment within Bering Strait School District.
	-Communications Outreach Specialist -Community Members at large -Lead Parent Stakeholder -Lead Patient Partner -Lead Audiology Stakeholder	• Facilitated development of a region-specific addendum to the hearing quality of life measure (HEAR-QL). • Developed measures to address the sensitivity of the sociodemographic survey, including an informational cover sheet.
**Telemedicine Protocols**	-Community Members at large	• Responded with mixed preferences about being present for a child’s initial follow-up appointment. Some parents preferred to be there; others preferred not. Both of these preferences were built into the intervention process.
	-Lead Audiology Stakeholder -Village-based Healthcare Providers -NSHC Village Health Services Administration	• Built new workflows for telemedicine cases to be completed by CHA/Ps; developed processes for scheduling and blocking CHA/P availability.
**Participant Recruitment & Retention**	-Communications Outreach Specialist	• Designed and led all the social media, announcements, flyers, and communication to communities. Assisted with in-person communication and enrollment and collaborated with the schools to help disseminate and collect forms.
	-Lead Education Stakeholder	• Provided essential leadership with recruitment throughout the school district, emphasizing the value of the study in contributing to students arriving to the classroom ready to learn.
	-Lead Parent Stakeholder	• Participated in and assisted with community events and focus groups. Provided insight as a parent, a patient, and a community member, and assisted with communications to members of the community about the project. Reviewed and edited media blasts before their release and provided input during weekly meetings during the recruitment phase.
	-Community Members at large -Lead Audiology Stakeholder	• Provided real-time feedback that encouraged the use of FM radio, VHF radio, Facebook, flyer, and word of mouth forrecruitment. Offered input on decisions about location and format of public forums, or focus groups.
	-School staff	• Collaborated with team in order to get necessary paperwork in place for children participate in the study.

### Research question development

For years before the start of the trial, the Lead Surgeon Stakeholder and the Lead Hospital Administration Stakeholder had observed cases in which children with hearing loss were identified and treated much later than necessary, due to a lack of follow-up care. These individuals shared their observations with an otolaryngologist/public health researcher who had experience working in Alaska and a practicing NSHC audiologist. After initial discussions, this group brought their observations to individuals who would become the Lead Education Stakeholder and Lead Parent Stakeholder. Collectively, this group determined that linking the existing school hearing screenings to the statewide telemedicine network could represent a feasible intervention to ensure that children with hearing loss would be efficiently connected to the health care system for diagnosis and treatment. Telemedicine capabilities at community health clinics had never before been applied for preventive measures such as school hearing screenings.

The otolaryngologist/public health researcher led the scientific team and recruited individuals with complementary research expertise, while the Lead Audiology Stakeholder, Hospital Administrator, and Parent Stakeholders recruited additional stakeholders during the formation of the study team (Table 1). The study team developed a proposal and was awarded funding from the Patient-Centered Outcomes Research Institute (PCORI).

### Comparators for screening processes

The scientific team sought to compare the current school screening protocol to an alternative mHealth protocol. The mHealth protocol, which included a validated cell phone-based screening and tympanometry, a measure of middle ear function that is important for detection of ear infections, was selected by the principal investigators (the Lead Audiology Stakeholder and the otolaryngologist/public health researcher). Selection was based on consideration of systematic reviews [31] and regional prevalence data [7–9], as well as feedback from the Lead Education Stakeholder and the teacher stakeholder focus group, both of which emphasized the need to prioritize ease of use and affordability. The Alaska stakeholder team advocated for all enrolled children to benefit from receiving all screening protocols, rather than randomization to current school screening or mHealth screening. Therefore, the study team chose not to randomize screening protocols. All enrolled children in all 15 communities would undergo the same three hearing evaluations: the current school screening protocol, mHealth screening, and an audiometric evaluation. The scientific team and the Alaska stakeholder team agreed to measure the sensitivity and specificity of the mHealth screening and current school screening protocols, compared to a benchmark audiometric evaluation.

### Comparators for referral processes

The scientific team and Alaska stakeholder team sought to compare the existing standard primary care referral pathway to a telemedicine specialty referral pathway. The Alaska stakeholder team planned for the study to generate a referral list after the school screenings in each community and transfer this list to school leadership and clinic staff, who would manage the referral process. In the telemedicine specialty referral pathway, CHA/Ps would conduct store-and-forward telemedicine consults containing vitals, case history, and basic testing, such as images of the ears and tympanometry. This information was forwarded to specialists for asynchronous review. If surgical or medical management were needed, the receiving audiologist would forward to an otolaryngologist for further consultation. In the standard primary care referral pathway, a letter would be sent home for children requiring referral to inform the parents/caregivers of the referral and to ask them to contact the clinic to set up a follow-up appointment. This letter notification had been standard practice for BSSD for many years. Despite anecdotal observations that children were often lost to follow-up with this process, the standard primary care referral process had never been evaluated.

### Unit of randomization

The scientific team proposed randomization as the most scientifically rigorous methodology for comparing the standard primary care referral pathway to the telemedicine specialty referral pathway. The Alaska stakeholder team advised that randomization at the community level, rather than individual, would be most appropriate and feasible within the regional context of living in small isolated rural communities. The Communications Outreach Specialist and Lead Parent Stakeholder guided the scientific team through the process of building strata for randomization based on geographic and cultural considerations across the 15 communities.

### Choice of outcomes and measures

The primary outcome of the study was time to first ear/hearing International Statistical Classification of Diseases, 10th Revision (ICD-10) diagnosis, measured in days from the date of school screening. Secondary outcomes included sensitivity and specificity of screening protocols compared to a benchmark audiometric evaluation, prevalence of hearing loss, hearing-related quality of life, and school performance.

To assess academic performance, the Lead Education Stakeholder directed the team to use AIMSweb, which was the standard benchmark assessment for BSSD at the time. For the quality of life measure, the scientific team proposed using Hearing-Related Quality of Life (HEAR-QL) questionnaire, the only validated instrument to measure hearing quality of life in children [6]. The scientific team brought the questionnaire to the Alaska stakeholder team for input and modifications. The Lead Parent Stakeholder and the Lead Patient Partner facilitated discussions about the questionnaire, and community members were asked for feedback during focus groups. One parent shared that when their son was young, he was sensitive to the loud noises in the school gym, particularly buzzers. They suggested that, since some of the questionnaire focused on environments, questions might be added to specifically include noisy situations commonly encountered in their communities. Given the popularity of community events held in the school gym, including basketball games and traditional dancing and drumming events, the Lead Parent Stakeholder recommended adding a question about noise in the gym. Additionally, a parent specifically suggested scenarios related to subsistence activities: “Do you not like to go hunting because it’s too loud? Do you not like to ride four wheelers, boats … (?)” Given this feedback, supplementary questions on these topics were added as a region-specific addendum to the HEAR-QL questionnaire.

A 14-question sociodemographic survey was developed to gather information about the household environments of children enrolled in the study. The survey was based on a questionnaire that had been used in research in western Alaska to assess environmental risk factors in relation to respiratory infections [32]. During focus groups, facilitators showed participants the questionnaire and asked if it would be acceptable for the team to collect this kind of information. Comments received were non-specific but affirmative: *“Yeah, they ask that on other applications. I don’t see why you guys can’t …*” When the facilitator probed about the content on these other applications, a participant shared that these forms inquire about “*… The same stuff you know. Income. Just the same things you mentioned. Real straightforward.”* General feedback on formatting was received, such as *“Try not to use big words in your surveys.”* The Lead Parent Stakeholder, Lead Patient Partner, and the Communications Outreach Specialist built upon this feedback by reviewing several iterations of survey drafts, and by advising the study team on specifically how to modify the survey’s language around sensitive topics (e.g. income, running water, smoking practices in the home). An informational cover sheet was also developed with Alaska stakeholder team involvement, and was attached to the questionnaires to clarify the intent in a few short sentences.

### Telemedicine protocols

#### Communication and parent involvement

In focus groups with parents and community members, there was extensive discussion over whether caregivers preferred to be present if their children needed to be seen for telemedicine specialty referral in the clinic. Opinions expressed during focus groups were variable and contradictory. Some thought that the follow-up should happen regardless of parent availability and presence. *“That’s what I was going to mention, the parents really don’t have to be there after the school screening. I think it’s more important for kids to be able to hear rather than have their parents there. Because there’s learning problems due to hearing.”* Others expressed the desire to be present. *“Yes I want to be there in person, the day of. Because I think that as parents we should always be with our children when there is [sic] medical practices being done.”* Given the conflicting feedback, the study team designed a process that would accommodate divergent preferences. At the beginning of the school year, parents were offered the option to sign a consent form so that their child could be seen for a telemedicine appointment without them present, in an effort to reduce barriers to care. In the event that the child required referral, clinic staff would make a phone call to notify the parents about their child’s follow-up and give them the option of accompanying the child. In a focus group, a participant advised that *“… for the younger grades you might consider a different process because a three or four-year-old who’s found in ECE [Early Childhood Education] coming in by themselves from the school wouldn’t work without a parent.”* In accordance with this advice, for children 2nd grade and below, clinic staff would ask parents to accompany their child. The phone call to the parent and the age-based strategy was based specifically on this feedback obtained during focus groups.

#### Design of telemedicine workflow

The Lead Audiology Stakeholder led efforts to incorporate telemedicine specialty referral appointments into the clinics’ workflow, collaborating across several media with CHA/Ps, Supervisor/Instructors (SIs), the Director of Village Health Services (VHS), the Vice President of VHS, and the Billing and Medical Records departments at NSHC. In a focus group, CHA/Ps and mid-level community-based providers shared their feedback on the weaknesses of telemedicine from their perspective. *“It takes too much time. You gotta copy and scan it and then you gotta do the telemed. To do that, it takes time away from seeing other patients, and dealing with other things. The telemed causes too many deficiencies … If we don’t get another co-signature right away from another provider, we get the deficiencies saying we’re not doing our jobs right. Like with the documentation.”* Given this and other feedback regarding workload burden for CHA/Ps, the Lead Audiology Stakeholder developed a new CHA/P workflow for the telemedicine appointments. In this workflow, patient history and exam forms were condensed to accommodate the CHA/Ps’ needs for quicker cases in busy clinics. This design incorporated feedback from Medical Records and Billing to ensure encounters were billable to facilitate sustainability. To further lessen the burden on CHA/P time and resources, chart review responsibilities were moved to the audiologist consulting on the telemedicine referral.

### Participant recruitment and retention

The Communications Outreach Specialist led efforts to publicize the study through appropriate mediums in order to maximize participation in the community focus groups. Mediums for recruitment were based on real-time feedback from stakeholders and community members, who encouraged the use of FM radio, VHF radio, community-wide social media, flyers, and word of mouth. The Alaska stakeholder team advised on decisions about location and format of focus groups. The project name was created by the Communications Outreach Specialist and the logo, which was used as branding on all printed and web formats, was designed by a local artist.

Feedback from the community events and focus groups guided recruitment for the trial. The Lead Parent Stakeholder, Lead Patient Partner, and Communications Outreach Specialist helped to translate themes from the focus groups and events into actual recruitment strategies and study communication. The Communications Outreach Specialist assisted with in-person trial enrollment and collaborated with schools to disseminate and collect forms. Additionally, the Communications Outreach Specialist developed an infographic flyer to communicate the purpose of the study through a visual medium and was attached to consent forms. The Lead Education Stakeholder provided essential leadership with recruitment, engaging staff across BSSD, and emphasizing the value of the screenings. As the Coordinator of Special Education for the district, this stakeholder was a critical connection to Special Education teachers, who typically conduct the school hearing screenings and who would be integrally involved in the data collection process. Meanwhile, the Lead Parent Stakeholder provided invaluable perspectives as a parent, health system user, and community member, assisting with study-related communications to community members, reviewing and editing social media announcements, and providing input during weekly meetings in the recruitment phase. The Lead Parent Stakeholder also actively assisted with community events and focus groups in the region.

## Discussion

Collaborative and community-based processes are crucial ‘best practices’ within health research that involves Alaska Native communities. Alaska Native communities have endured colonization and marginalization, some of which has been perpetuated through unethical research [23, 33, 34]. Research built upon community involvement is widely called for within the literature to prevent the perpetuation of colonialist dynamics between researchers and the researched [21–23, 35]. Several studies conducted within Alaska Native or American Indian communities have highlighted community involvement as central to the success or sustainability of research programs [25, 36]. However, few studies transparently report on community involvement activities that shape the design of health research in Indigenous communities [22, 24, 25], particularly in the Circumpolar North [23]. This paper summarizes the process of community participation and stakeholder involvement that informed the development of a community randomized trial in 15 rural Alaska Native communities.

The Hearing Norton Sound community randomized trial was built around feedback gathered through one-time engagement and participation in community events and focus groups, and continuous community involvement with the Alaska stakeholder team working in partnership with the scientific team. This diverse level of community involvement was essential. The Alaska Native Health Research Forum has advocated for the incorporation of “multiple, flexible, and community-driven points” for effective engagement [37]. In the Hearing Norton Sound study, community events and focus groups provided an avenue through which a range of cross-sectoral individuals and lay community members could participate and share feedback. Meanwhile, the Alaska stakeholder team partnered with the scientific team to provide iterative feedback that refined and finalized the suggestions from focus groups and other one-time engagements. While it is difficult to assess the overall effectiveness of community involvement strategies [38], we can consider the ways these strategies concretely facilitated community guidance over the research process as indicators of their success (Table 2).

### Limitations

Despite involvement of key local stakeholders on the study team and broad outreach across the region in the form of community events and focus groups, perspectives are limited to the individuals that participated and may not represent the entire region. By employing multiple modalities of involvement, the Hearing Norton Sound study team attempted to mitigate these limitations. In particular, the inclusion of one-time community events and focus groups open to the public allowed us to offer a level of involvement which posed a lesser time commitment for participants.

In some focus groups, certain topics of discussion generated substantial conversation and constructive feedback, but in others, responses to the facilitators’ questions were less robust. The large size of some focus groups may have contributed to this limitation. Additional factors, such as competing priorities in participants’ daily lives or unfamiliarity with providing feedback on research design could have also contributed. These factors compound barriers that have been identified elsewhere in the literature on engagement with Alaska Native communities, such as difficulties communicating with remote and distinct communities across vast geographical regions, and distrust of research due to previous negative experiences [21]. To increase participation, the Communications Outreach Specialist and Lead Parent Stakeholder, who are from the region, moderated the focus groups alongside the local Lead Audiology Stakeholder and Lead Hospital Administration Stakeholder, and iteratively adjusted language around certain questions. To address potential discomfort around research, focus group moderators took time at the beginning of each event to explain the importance and intentions of community involvement in research and the value of feedback, experiences, and insight of all types in informing the research project.

Lastly, it is important to acknowledge the potentially conflicting role of a local stakeholder who is also rooted in the research process. However, we found this to be a strength through which the study was grounded in local culture and perspectives. In particular, the Lead Audiology Stakeholder was well-versed both in the local environment and also research methodology. This facilitated effective communication between the ‘lay’ individuals on the team and the academic scientists.

## Conclusions

Teamwork and iterative community involvement influenced the design of the Hearing Norton Sound trial at numerous key decision points and facilitated listening to community members through both one-time participation and continuous involvement. The authenticity of stakeholders’ feedback relied on their positionality within their respective sectors, and their stake in the belief that the research would provide a foundation for better hearing services for children in rural Alaska Native communities. Hearing Norton Sound offers an example of multifaceted stakeholder involvement in study design that provides an example for future research conducted within Alaska Native communities.

## Supplementary information

**Additional file 1.** Guidance for Reporting Involvement of Patients and the Public Long Form. This file includes a completed GRIPP Long Form Checklist to be used when reporting community involvement and engagement.

## Data Availability

The datasets (qualitative transcripts and notes) analyzed during the current study are not publicly available due to potential compromise in individual and community privacy but are available from the corresponding author on reasonable request and with approval from the institutional review boards of Alaska Area and Norton Sound prior to release of any de-identified or limited datasets.

## Abbreviations

NSHC: Norton Sound Health Corporation
CHA/Ps: Community health aides/practitioners
BSSD: Bering Strait School District
HEAR-QL: Hearing-related quality of life
SI: Supervisor Instructor
VHS: Village Health Services
ICD-10: International Statistical Classification of Diseases, 10th Revision
